# Microbiome–Metabolomic Analysis Reveals Beneficial Effects of Dietary Kelp Resistant Starch on Intestinal Functions of Hybrid Snakeheads (*Channa maculata* ♀ × *Channa argus* ♂)

**DOI:** 10.3390/antiox12081631

**Published:** 2023-08-18

**Authors:** Shaodan Wang, Zhiheng Zuo, Bin Ye, Li Zhang, Yanbo Cheng, Shaolin Xie, Jixing Zou, Guohuan Xu

**Affiliations:** 1 State Key Laboratory of Applied Microbiology Southern China, Guangdong Provincial Key Laboratory of Microbial Culture Collection and Application, Institute of Microbiology, Guangdong Academy of Sciences, Guangzhou 510070, China; 20201067007@stu.scau.edu.cn (S.W.); zhl9813@163.com (L.Z.); huaqingzq@163.com (Y.C.); 2 Joint Laboratory of Guangdong Province and Hong Kong Region on Marine Bioresource Conservation and Exploitation, College of Marine Sciences, South China Agricultural University, Guangzhou 510642, China; zuozhiheng@stu.scau.edu.cn (Z.Z.); yebinjob@163.com (B.Y.); xieshaolinscau@163.com (S.X.)

**Keywords:** resistant starch, microbiome, metabolomics, intestinal morphology, intestinal function

## Abstract

The benefits of resistant starch on hypoglycemia, obesity prevention, antioxidant status and the alleviation of metabolic syndrome have received considerable attention. In this study, we explored how dietary kelp resistant starch (KRS) enhances intestinal morphology and function through a microbiome–metabolomic analysis. Hybrid snakeheads (initial weight: 11.4 ± 0.15 g) were fed experimental diets for 60 days. Fish were fed a basic wheat starch diet and the KRS diet. Dietary KRS improved intestinal morphology and enhanced intestinal antioxidant and digestive capabilities, as evidenced by decreased intestinal damage and upregulated intestinal biochemical markers. The microbiome analysis showed that KRS administration elevated the proportion of butyrate-producing bacteria and the abundance of beneficial bacteria that increases insulin sensitivity. Furthermore, significant alterations in metabolic profiles were observed to mainly associate with the amino acid metabolism (particularly arginine production), the metabolism of cofactors and vitamins, fat metabolism, glutathione metabolism, and the biosynthesis of other secondary metabolites. Additionally, alterations in intestinal microbiota composition were significantly associated with metabolites. Collectively, changes in intestinal microbiota and metabolite profiles produced by the replacement of common starch with dietary KRS appears to play an important role in the development of intestinal metabolism, thus leading to improved intestinal function and homeostasis.

## 1. Introduction

Carbohydrates, lipids, and proteins are the major sources of energy for the life of vertebrates. In contrast to other animals that use carbohydrates as the main energy source, fish primarily rely on proteins and lipids for energy [1]. The drawback of using protein of animal origin as the primary source of protein in fish diets is its high price. Animal protein can be replaced with vegetable protein, but the main disadvantage of the latter is that it contains anti-nutritional factors. Carbohydrates are regarded as the cheapest energy source, which can efficiently reduce the need for fish to feed on protein through rational usage [2]. However, dietary carbohydrates frequently result in an increase in blood sugar levels in fish, especially carnivorous fish, since the carbohydrates in fish diets are digestible but difficult to utilize properly [3,4]. The developmental rates of fish can be impacted by long-term high blood sugar levels, which can also cause insulin resistance, fatty liver, and intestinal disorders [5,6].

Resistant starch (RS), known as a dietary prebiotic, is considered a possible alternative to subtherapeutic antibiotics, which are frequently employed to promote animal health and intestinal homeostasis [7,8]. RS is one of the complex carbohydrates that, due to its formation as a β-glycosidic bond, is comparatively resistant to host-produced α-amylases in the small intestine [9]. Because the upper gastrointestinal system cannot decompose RS, most of it can reach the colon, where lives in a large number of intestinal microorganisms. Complex carbohydrates in dietary components can be fermented by intestinal microorganisms to create beneficial metabolites like short-chain fatty acids (SCFAs) [10,11]. In addition, colonic epithelial cells utilize the metabolites generated by intestinal bacteria to supply energy and reduce the risk of insulin resistance [12,13]. The composition of intestinal microbes, such as the bacterial abundance and dominating species of Firmicutes and Bacteroidetes, is also affected by resistant-starch-enriched chyme [14]. Furthermore, the occurrence of intestinal damage and the function of the intestinal barrier are linked to the metabolites of intestinal microbes.

Kelp (*L. japonica*) is one of the most widespread brown seaweeds in the world. Studies have revealed that kelp polysaccharides have a variety of biological activities, including anti-tumor, anti-obesity, and anti-oxidation, decreasing blood lipids, and improving hyperglycemia [15,16]. The hypoglycemic impact of kelp polysaccharides is related to its resistant starch structural properties, which may resist host amylase degradation and slow the decomposition of ordinary starch [17,18]. Furthermore, by manipulating the host intestinal microbes, kelp polysaccharides can benefit intestinal health. For instance, kelp polysaccharides reduce metabolic abnormalities linked to obesity by modulating intestinal microbes [19]. However, it remains unclear whether resistant starch can ameliorate fish hyperglycemia caused by dietary starch and regulate the intestinal health of fish.

In this study, resistant starch (kelp meal) was used to partially or totally replace conventional starch in the diet of carnivorous fish, hybrid snakeheads (*Channa maculata* ♀ × *Channa argus* ♂), in order to examine the effect and mechanism of KRS on fish intestinal health. The relationship between changes in intestinal microbes and their metabolites and intestinal health was also investigated through conjunctional analyses of the intestinal microbiome and metabolome.

## 2. Materials and Methods

### 2.1. Experimental Diets

Dietary proteins consisted primarily of fishmeal, casein, and soybean meals, while dietary fats were primarily fish oil. The basic carbon source for the control group (C) was 20% high-gluten wheat flour, while in the experimental group, resistant starch (kelp meal, *Laminaria japonica*) was employed as an alternate carbon source. The KRS was dehydrated and dried from fresh kelp, ground into powder, and sterilized under a high temperature. Fish were allocated into three dietary groups based on the addition ratio of wheat flour and kelp meal: the control group (C, 20% wheat flour), the medium replacement group (MR, 10% wheat flour and 10% KRS), and the full replacement group (FR, 0% wheat flour and 15% KRS) (Table 1). All raw materials (Table S1) were ultra-finely pulverized and then uniformly mixed through a 100-mesh screen. The extruded feed manufacturing line offered by New Hope Company (Guangzhou, China) creates extruded pellets with a particle size of 2–2.5 mm. The prepared feeds of each group were extruded feeds with an equal amount of nitrogen and fat, and the FR group contained 15% KRS and 5% binder, which was easy to pelletize during the extrusion process.

### 2.2. Experimental Fish and Design

The experimental animals were healthy and high-quality juvenile hybrid snakeheads (*Channa maculata* ♀ × *Channa argus* ♂) (Yunfu, Guangdong), which are carnivorous fish, and were provided by the Xinxing fry hatching center. The culture experiment used a circular tank of 1000 L as the culture bucket and was conducted in a land-based recirculating aquaculture system. The animals were acclimated to the basal diet for two weeks before the formal trial in order for them to get used to the diet. After being weighed, identical-sized fish (11.4 ± 0.15 g) were chosen and randomly allocated into three groups, with three replicates in each group (90 fish per replicate). The ratio of male to female was close to 1:1.

The experimental fish were fed twice a day (8:00 and 16:00, 3–5% of body weight), and the experimental period was 60 days. During the experiment, the environmental conditions were maintained at a water temperature of 25–29 °C, dissolved oxygen level of >4 mg/L, and pH of around 7.

After fasting for 24 h, 9 fish were selected at random from each group. Following anesthesia with 3-Aminobenzoic acid ethyl ester methanesulfonate (MS-222, 100 mg/L) (6-0009, Tianjingsha, China), blood was collected from the tail vein, and the serum was extracted by centrifuging the blood at 4 °C at 4000 rpm. Following the blood sample, the midintestinal was rapidly removed for histopathological and biochemical analyses, and the hindintestinal and intestinal contents were obtained for investigation of the intestinal microbiome and metabolome. All samples, except those for histopathological analysis, were immediately frozen in liquid nitrogen and kept at −80 °C until analysis.

### 2.3. Serum Biochemical Analysis

Frozen serum was thawed on ice. According to the manufacturer’s recommendations, the serum total cholesterol (T-CHO, A111-1-1), triglyceride (TAG, A110-1-1), and glucose (Glu) levels were determined by using a test kit (F006-1-1, Nanjing Jiancheng, Bioengineering Institute, Nanjing, China).

### 2.4. Intestinal Histopathological Analysis

Paraffin slices were used to view morphological changes in the intestinal tissue; see Table S2 for details. Fresh tissues were immersed in a 4% paraformaldehyde solution (E672002, Sangon Biotech, Shanghai, China) for seven days, dried, embedded in paraffin, sectioned (4 μm), and stained (hematoxylin and eosin staining) (G1005, Servicebio, Wuhan, China). The M8 automated digital scanning imaging system (Wanbangjunyi, Beijing, China) was used to observe and photograph the paraffin slices. The data were measured using the image-pro plus 6.0 by two observers who were unaware of the study’s design.

### 2.5. Intestinal Biochemical Analysis

The cryopreserved intestinal tissue was thawed on ice and processed into a 10% tissue homogenate (*w*/*v*: 1/9) using 0.9% normal saline for biochemical activity tests. Intestinal oxidative stress indicators were measured to determine intestinal health: superoxide dismutase (SOD, A001-3), malondialdehyde (MDA, A003-1), catalase (CAT, A007-1-1), and glutathione peroxidase (GSH-Px, A005-1). The digestive enzymes alpha-chymotrypsin (α-Chmo, A080-3-1), alpha-amylase (α-Ams, C016-1-1), and lipase (Lip, A054-2-1) were also evaluated. Alkaline phosphatase (AKP, A059-2) measures the effectiveness of the intestinal mucosal barrier. All enzymes were tested using appropriate commercial kits (Nanjing Jiancheng, Bioengineering Institute, Nanjing, China), and the protein level in the sample was examined to determine the relevant enzymatic activity. Intestinal monocarboxylate transporter-1 (MCT-1, FY69708-B) was detected using an ELISA kit (Nanjing Caobenyuan, Biotechnology Co., Ltd., Nanjing, China).

### 2.6. Intestinal Microbiome Analysis

A FastDNA™ Spin Kit for Plant and Animals Tissues (MP Biomedicals, Shanghai, China) was used to extract the intestinal microbiome’s genomic DNA in accordance with the manufacturer’s instructions. The V3–V4 regions of 16S rDNA (338F: 5′-TCCTACGGGAGGCAGCAG-3′ and 806R: 5′-GGACTACHVGGGTWTCTAAT-3′) were sequenced and analyzed using Illumina MiSeq-PE250, and MiSeq libraries were constructed. The pair-end reads obtained via sequencing were aligned and assembled using FLASH (v1.2.11). Chimeric sequences were then identified using the software UPARSE (v7.0.1090), USEARCH (v11), and UCHIME (v4.2.40), and non-repetitive sequences were clustered into operational taxonomic units (OTUs) based on a 97% similarity threshold.

Based on the Qiime platform, the RDP classifier (v2.11) Bayesian algorithm was used to perform taxonomic analysis on the representative sequences of OTUs with a similar level of 97%. Intestinal microbial diversity was evaluated using an alpha-diversity analysis, while sample species differences were evaluated using a beta-diversity analysis. The evaluation of bacterial groups with substantial differences was performed using the LEfSe multilevel species difference discriminant analysis. PICRUSt was used for the predictive analysis of the roles of differential genes.

### 2.7. Metabolomic Analysis

Liquid chromatography–mass spectrometry (LC-MS)/MS technology was used to analyze the metabolic profiling of intestinal content (Majorbio, Shanghai, China). For metabolite extraction, a 400 L methanol–water extract (4:1 *v*/*v*) with 0.02 mg/mL of internal standard (L-2-chlorophenylalanin) was added to a 100 mg intestinal sample. Following 30 min of low-temperature ultrasonic extraction, the samples were placed at −20 °C for 30 min before being centrifuged at 13,000 rpm for 15 min at 4 °C to obtain the supernatant for analysis. In parallel, an equal volume of each sample’s metabolites was combined to create a quality control sample (QC), which was then injected into the analysis to verify repeatability.

The HSS T3 column (100 mm × 2.1 mm i.d., 1.8 μm) was used to separate 2 μL of the sample before it underwent mass spectrometry detection. The mobile phases were composed of 0.1% formic acid in acetonitrile/water (solvent A) at a ratio of 95:5 and 0.1% formic acid in acetonitrile/isopropanol/water (47.5:47.5:5, *v*/*v*), respectively (solvent B). Positive and negative ion operating modes were used to acquire mass spectral data (3500 V for positive ions; −2800 V for negative ions) and normalized collision energies (20–40–60 V cycle). The resolution of MS/MS was 17,500, while the full MS resolution was 70,000. Mass spectral data were carried out over a mass range of 70–1050 m/z.

Progenesis QI software (v3.0, Waters Inc., Milford, MA, USA) was used to evaluate the raw UPLC-MS data, and the Majorbio I-Sanger Cloud Platform was used to aggregate and analyze the positive and negative data (www.i-sanger.com, accessed on 11 January 2022). The Metabolite and Tandem MS Database (METLIN), Human Metabolome Database (HMDB), and Majorbio Database were used to identify the metabolites. Principal Component Analysis (PCA) and Orthogonal Least Partial Squares Discriminant Analysis were carried out using R software (v1.6.2) (OPLS-DA). KEGG database annotation of metabolic pathways was used to identify pathways involved in various metabolites.

### 2.8. Statistical Analysis

Excel, image-pro plus 6.0, GraphPad Prism, and SPSS 20 were used for statistical analysis and the charting of the data. A one-way ANOVA analysis of variance and Duncan’s method for multiple comparisons (*p* < 0.05) were used to identify the differences in the data.

## 3. Results

### 3.1. Regulation of KRS on Blood Glucose and Blood Lipids

**Figure 1:**
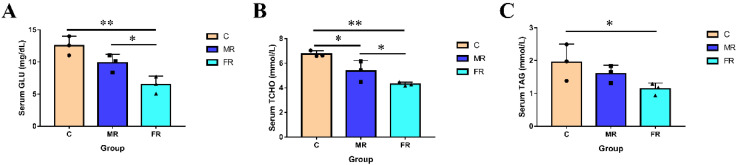
Regulation of KRS on serum glycolipid levels in hybrid snakeheads. (**A**) Serum glucose levels. (**B**) Serum total cholesterol content. (**C**) Serum triglyceride content. Values are expressed as means ± SD. * Indicates significant difference (* *p* < 0.05, ** *p* < 0.01).

The serum parameters of each group are shown in Figure 1. As a carbon source, KRS lowered serum Glu, T-CHO, and TAG levels. In comparison to those in group C, the concentration of serum Glu and T-CHO dropped with the increase in KRS levels and was the highest in the FR group (*p* < 0.01). Serum TAG levels in FR were also significantly lower than those in the control group C (*p* < 0.05). The results indicate that the replacement of conventional starch with KRS as a carbon source can lower blood sugar and blood lipids in hybrid snakeheads.

### 3.2. KRS Favors Intestinal Villi Growth and Reduces Villi Damage

**Figure 2:**
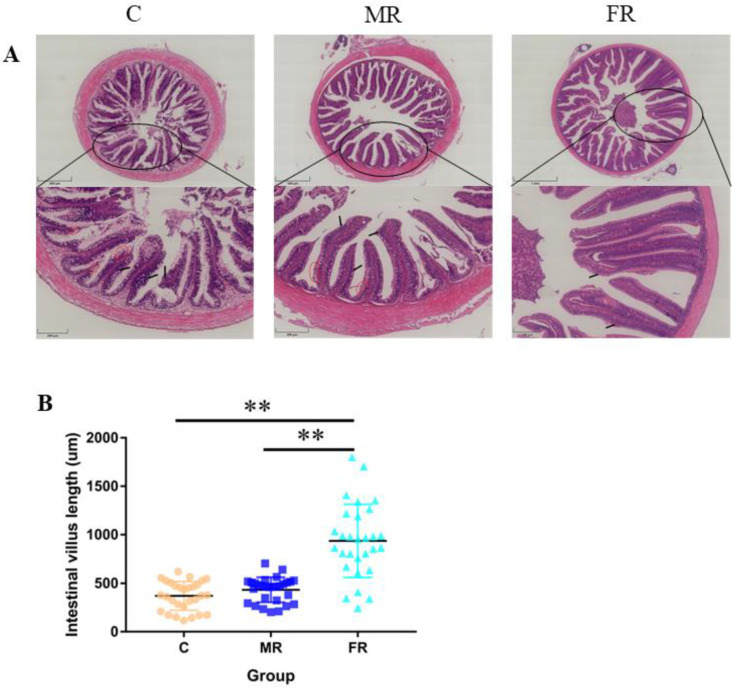
Intestinal villi growth and intestinal damage. (**A**) H&E staining of the midintestinal portion of the intestine. Upper scale bar = 500 μm, below scale bar = 200 μm. (**B**) Intestinal villus length statistics. The red circle highlights the shedding of the intestinal villus epithelium. Values are expressed as means ± SD. * Indicates significant difference (** *p* < 0.01).

Intestinal tissue was stained with H&E to reveal that the high-carbohydrate diet-fed control group experienced considerable intestinal villi damage and intestinal villus epithelium shedding (Figure 2A). The average length of the intestinal villi in the FR group considerably increased in comparison to that in the C group (*p* < 0.01) (Figure 2B), suggesting that the use of resistance starch could reduce intestinal damage and promote intestinal villi formation.

### 3.3. KRS Has the Potential to Improve Intestine Antioxidant Function, Digestion and Absorption, and Mucosal Barrier Function

**Figure 3:**
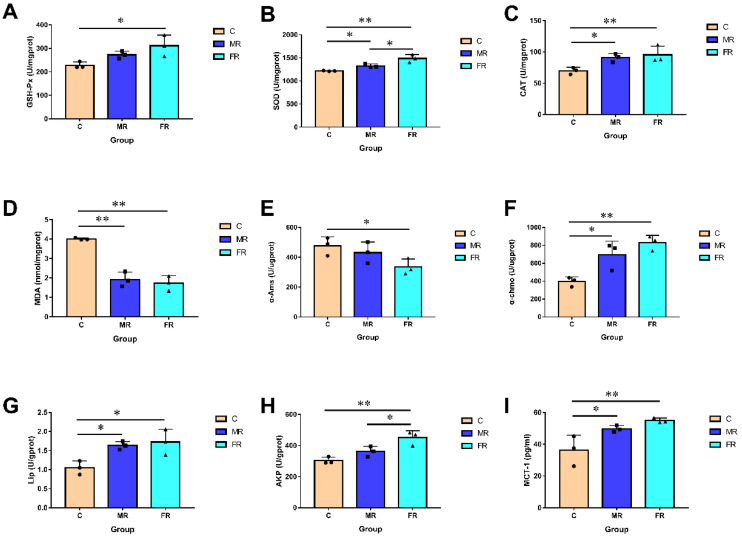
KRS affects intestinal biochemical activity. (**A**) Intestinal GSH-Px activity. (**B**) Intestinal SOD activity. (**C**) Intestinal CAT activity. (**D**) Intestinal MDA activity. (**E**) Intestinal α-Ams activity. (**F**) Intestinal α-Chmo activity. (**G**) Intestinal Lip activity. (**H**) Intestinal AKP activity. (**I**) Intestinal MCT-1 activity. Values are expressed as means ± SD. * Indicates significant difference (* *p* < 0.05, ** *p* < 0.01).

The morphology and health of the intestinal villi can directly affect intestinal function. Since intestinal injuries can induce oxidative stress, we examined the activity of intestinal antioxidant enzymes. The GSH-Px, SOD, and CAT activities of the FR group were considerably higher than those of the C group (*p* < 0.01) (Figure 3A–C). This implies that, in contrast to a high-carbohydrate diet, KRS has no negative effects on the intestinal antioxidant capacity. KRS appeared to reduce the oxidative stress in the intestinal villi since the SOD and CAT activities in the MR group were likewise noticeably higher than those in the C group (*p* < 0.05). Furthermore, the content of MDA (the final product of body peroxidation) in the MR and FR groups was significantly lower than that in the C group (*p* < 0.01) (Figure 3D).

We also assessed the activities of intestinal digestion enzymes. The results indicated that the MR and FR groups had significantly higher α-Chmo and Lip activity than the C group (*p* < 0.05) (Figure 3F,G). The α-Ams activity, however, exhibited a reverse pattern (*p* < 0.05) (Figure 3E). Additionally, as one of the crucial elements of the intestinal mucosal barrier function, the intestinal AKP activity was significantly enhanced in the MR and FR groups when compared to that in the C group (*p* < 0.05) (Figure 3H). In comparison with that in group C, MCT1 activity was significantly enhanced in MR and FR and was positively correlated with the dose of KRS (*p* < 0.05) (Figure 3I).

### 3.4. Modulation of Intestinal Microbial Composition via KRS at Different Dietary Carbohydrate Levels

**Figure 4:**
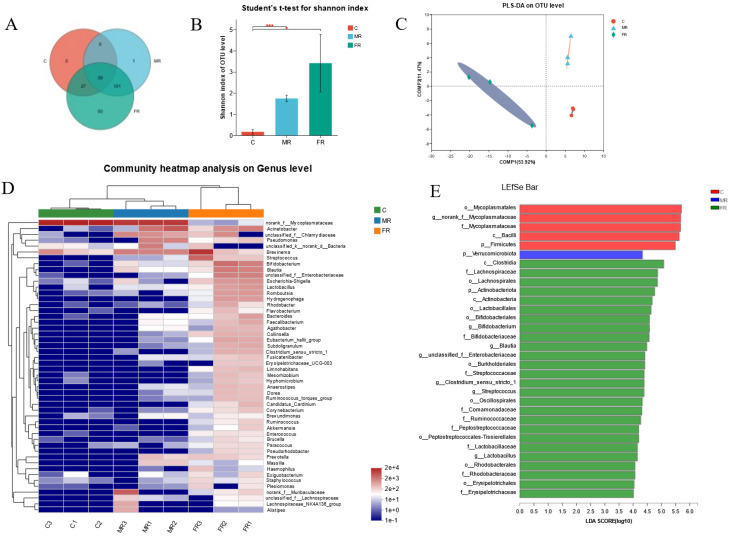
Dietary KRS modifies the composition of microbial population in the intestinal. (**A**) Venn diagram of intestinal differential microbes. (**B**) Shannon index of OTU levels. (**C**) Partial least squares discriminant analysis (PLS-DA) for differential grouping. (**D**) Genus-level species clustering heatmap for differential grouping. (**E**) Differential microbiome based on LEfSe analysis. Values are expressed as means ± SD. * Indicates significant difference (* *p* < 0.05, *** *p* < 0.001).

Intestinal microbiome profiling using 16S rRNA sequencing was performed to investigate the impact of dietary KRS on intestinal microbial composition. Among the 218 OTUs, the FR and MR groups increased by 152 OTUs compared with the C group, and 52 OTUs were unique to FR (Figure 4A). At the OTU level, α-diversity analyses were carried out in order to better comprehend changes in the community diversity and species richness of intestinal microbial communities. The Shannon index of the MR and FR groups was much higher than that of the C group (*p* < 0.05) (Figure 4B), whereas the Simpson index was significantly lower (*p* < 0.01) (Figure S1B), showing that KRS promoted community diversity. Meanwhile, in the MR and FR groups, the Chao1 index was much higher, indicating that the species richness was greater than that in the C group (*p* < 0.01) (Figure S1A). According to partial least squares discriminant analysis (PLS-DA), the samples from the MR and FR groups were significantly differentiated from the C group (Figure 4C). Principal coordinate analysis (PCoA) of intestinal microbials revealed that the FR and C groups were in separate quadrants, with significant species differences (PC1: 50.06%; PC2: 19.44%) (Figure S1C).

At the phylum level, Firmicutes, Spirochaetota, Proteobacteria, Verrucomicrobiota, Bacteroidota, and Actinobacteriota were the dominant species (Figure S1D). The top ten dominant species at the genus level were *norank_f__Mycoplasmataceae*, *Brevinema*, *Acinetobacter*, *norank_f__Muribaculaceae*, *Bifidobacterium*, *unclassified_f__Chlamydiaceae*, *Streptococcus*, *Blautia*, *Pseudomonas*, and *unclassified_f__Enterobacteriaceae* (Figure S1E). The clustering heat map reveals that the abundance of various intestinal microbial species varied between the MR group and the C group; however, there were also significant differences between the FR and the C group (Figure 4D). In addition, the species compositions of the MR and C groups were similar at the cluster level, whereas the FR group and C group were widely divided there (Figure 4D).

The Kruskal–Wallis H test was used in the grouped samples, and the phylum-level species Firmicutes were dramatically reduced in the MR and FR groups, while Proteobacteria, Actinobacteriota, Verrucomicrobiota, and Nitrospiota were greatly increased (*p* < 0.05) (Figure S2A). The genus-level species *norank_f__Mycoplasmataceae* decreased significantly in MR and FR groups, and other top ten species, such as *Bifidobacterium*, *Streptococcus*, *Blautia*, *unclassified_f__Enterobacteriaceae*, *Lactobacillus*, *Hydrogenophaga*, *Eubacterium hallii group*, *Collinsella,* and *Subdoligranulum*, were significantly increased in the MR and FR groups, and the FR group showed the most significant changes (*p* < 0.05) (Figure S2C). In addition, the LEfSe analysis (LDA: 4) indicated that the bacteria of the genera *norank_f__Mycoplasmataceae* and *Bifidobacterium* were the ones that differed mostly between the FR group and the C group (Figure 4E).

The functional prediction based on PICRUSt revealed that the functional pathways influenced by KRS in the differential microorganisms were primarily enriched in metabolic pathways, including amino acid metabolism, carbohydrate metabolism, lipid metabolism, and secondary metabolite metabolism (Figure S2B,D).

### 3.5. Effects of Dietary KRS on Intestinal Metabolite Profiles

**Figure 5:**
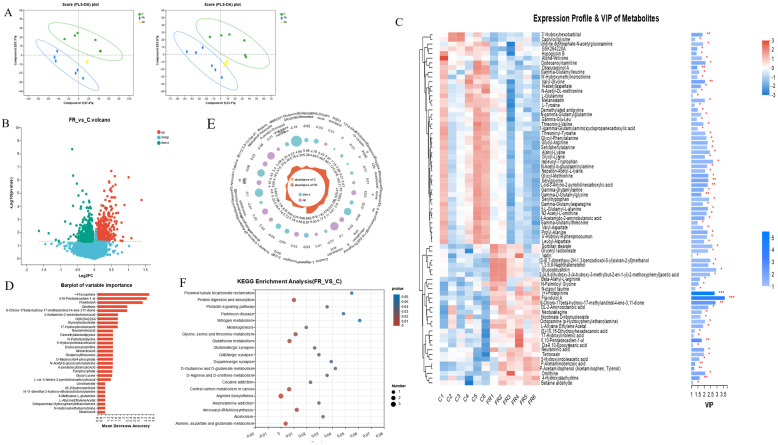
Characterization of intestinal metabolites in different groups. (**A**) Partial least squares discriminant analysis (PLS-DA) of intestinal metabolites in positive and negative ion mode. (**B**) Volcano plot of differential metabolites. (**C**) Cluster heatmap and VIP value analysis of differential metabolites. (**D**) The importance of differential metabolites was identified based on random forest plots. (**E**) Radar plots were used to visualize the expression profiles of various groups of differential metabolites. (**F**) KEGG analysis was used to examine the primary functions of differential metabolites. * indicates significant difference (* *p* < 0.05, ** *p* < 0.01, *** *p* < 0.001).

Based on the above findings, it was shown that intestinal morphology, intestinal function, and intestinal microbes showed the most significant differences between the C group and the FR group. To further investigate the impact of KRS on the intestine metabolic processes, metabolomic analysis was conducted to find the difference between the C group and the FR group. Partial least squares discriminant analysis (PLS-DA) showed a clear separation of metabolic profiles between the C and FR groups, and PC1 and PC2 demonstrated the model’s accuracy (positive ions: 47.4%, PC2: 10.1%; negative ions: 33.1%, PC2: 15.6%) (Figure 5A). Among all the identified metabolites, those with *p* < 0.05 and VIP > 1 were considered significantly differential metabolites. Volcano plots were used to depict all 1164 significantly differential metabolites. Based on random forest analysis, we identified key differential metabolites (Figure 5B). The annotated 75 differential metabolites and their expression levels and importance in various groups are shown in conjunction with the cluster analysis and VIP value of differential metabolites (Figure 5C). The following metabolites are associated with amino acid metabolism, such as +-prosopinine, ornithine, N-palmitoyl glycine, glutaminylthreonine, and glycyl-lysine, and the metabolism of fatty acids, such as FlavidulolA, 4-Acetamido-2-aminobutanoicacid, Glyceryllactoolate, 5-Hydroxyindoleaceticacid, Dodecanoylcarnitine, N-11-dimethyl-2-hydroxy-ethylarachidonoylamine, etc. (Figure 5D).

Further analysis found that 5-hydroxyindoleaceticacid, 2-aminoisobutyricacid, 13-Oxo-911-tridecadienoicacid, betaine, P-acetaminobenzoicacid, and 4-amino-2-methylenebutanoic acid significantly increased in the FR group, while glutaminylthreonine, cytidine, sakacinP, N-acetyl-L-glutamate5-semialdehyde, L-arginine, gamma-glutamylthreonine, and isoleucylproline were significantly reduced in the FR group (*p* < 0.05) (Figure 5E). The differentially abundant metabolites between the C group and the FR group were enriched on the KEGG pathways, including the metabolic pathways for amino acid metabolism (particularly arginine production), the metabolism of cofactors and vitamins, carbon metabolism, glutathione metabolism, and the biosynthesis of other secondary metabolites, which are all connected to the differentially abundant metabolites in the FR group (Figure 5F and Figure S3A). Through the association network of the KEGG pathway, we found that the metabolites including L-glutamine, L-tyrosine, and ornithine are associated with the most metabolic pathways (Figure S3B).

### 3.6. Correlation Analysis of Intestinal Microbial Communities and Metabolite Profiles

**Figure 6:**
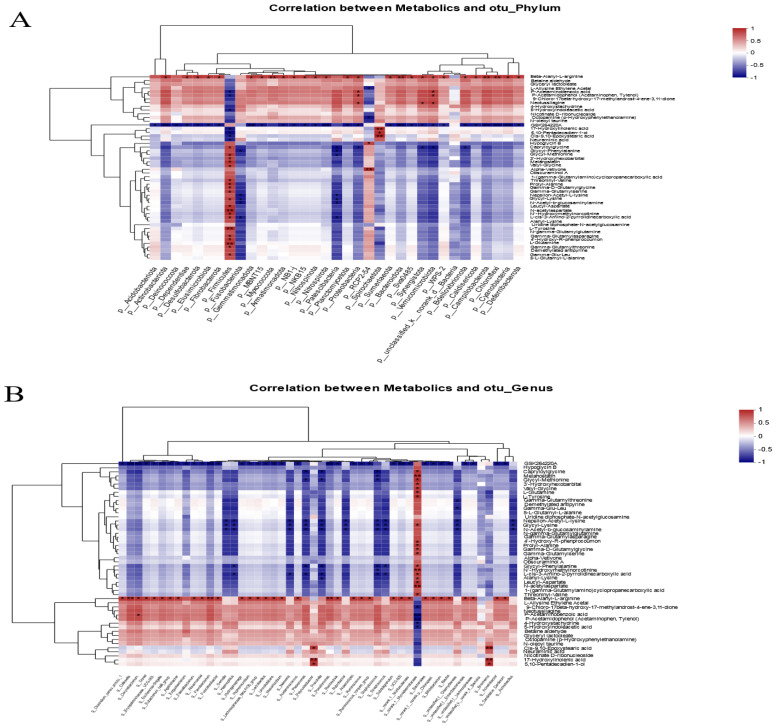
Heatmap of associations between intestinal microbial and metabolite profiles. (**A**) Pearson correlation analysis of phylum-level species and differential metabolites. (**B**) Pearson correlation analysis of genus-level species and differential metabolites. * indicates significant difference (* *p* < 0.05, ** *p* < 0.01).

Pearson correlation analysis was used to investigate the relationship between the *composition* of the intestinal microbiota and metabolites. The heatmap results showed that Firmicutes have a significantly positive correlation with N-acetylaspartate, L-tyrosine, and L-glutamine. Proteobacteria, actinobacteriota, and verrucomicrobiota were significantly increased in the FR group and were positively correlated with beta-alanyl-L-arginine, P-acetaminobenzoic acid, and P-acetamidophenol. Furthermore, beta-alanyl-L-arginine was negatively correlated with Firmicutes (*p* < 0.05) (Figure 6A). Further analysis found that *norank_f__Mycoplasmataceae* in Firmicutes were the main species associated with the production of differential metabolites. The *norank_f__Mycoplasmataceae* showed a significantly positive correlation with numerous amino acid metabolites such as L-glutamine, L-tyrosine, and N-acetylaspartate, and it was negatively correlated with fatty acid metabolites including P-acetaminobenzoic acid, P-acetamidophenol, and 5-hydroxyindoleacetic acid. In addition, *Bifidobacterium*, *Streptococcus*, *Blautia*, *unclassified_f__Enterobacteriaceae*, *Lactobacillus,* and other species enriched in the FR group were all positively correlated with beta-alanyl-L-arginine (*p* < 0.05) (Figure 6B). Therefore, it can be speculated that *norank_f__Mycoplasmataceae* and beta-alanyl-L-arginine are the main objects of the association between intestinal microbes and metabolites.

## 4. Discussion

The way to efficiently utilize carbohydrates in feed without endangering fish health is now one of the main problems in the aquaculture sector. It is commonly known that compared to herbivorous fish, carnivorous fish are inherent to diabetes. Dietary components involving carbohydrates will cause persistent hyperglycemia, which could result in issues like metabolic syndrome, insulin resistance, and fatty liver [20]. The reason for these health issues is the disturbance of intestinal flora and metabolites, which disrupt intestinal architecture and function [21,22]. However, a growing body of evidence shows that eating resistant starch improves intestinal health, obesity, and insulin resistance in experimental individuals [23,24,25]. Accordingly, we carried out a study on the replacement of starch with resistant starch (kelp meal) in the carnivorous fish hybrid snakehead in order to examine the benefits of KRS on the control of hyperglycemia as well as on intestinal microbial and metabolite profiles.

In general, prolonged post-meal hyperglycemia and the ensuing hyperlipidemia are the primary symptoms of poor carbohydrate metabolism [26]. In the present study, in contrast to group C fed with a high-carbohydrate diet, KRS did not result in post-prandial hyperglycemia. This is directly connected to the ability of resistant starch to absorb glucose and facilitate glucose diffusion while also resisting digestion by intestinal digestive enzymes [27]. Furthermore, in addition to causing high blood sugar, high-carbohydrate meals have been proven to be proportionate to the body’s lipid accumulation [28,29]. The KRS successfully lowered the serum T-CHO and TAG of the hybrid snakehead, which was advantageous in preventing lipid buildup and hyperlipidemia.

The digestive and absorption functions of the intestines are surely affected by a metabolic disorder [30]. In the intestine of the hybrid snakeheads with diets supplemented with KRS, higher levels of alpha-chymotrypsin and lipase activity were detected. Moreover, RS decreased the activity of α-Ams and slowed down the absorption and digestion of common starch constituents. According to studies, one of the key methods for treating and preventing hyperglycemia is reducing intestinal oxidative stress [16,31]. The activity levels of the antioxidant enzymes GSH-Px, SOD, and CAT in the MR and FR groups were substantially enhanced by KRS in a dose-dependent manner. In addition, the presence of KRS considerably reduced the activity of MDA, a marker of body peroxidation. Intestinal damage occurs when the tissue is under oxidative stress [18,32]. Intestinal injury impairs the function of the intestinal mucosal barrier and leads to intestinal cell death [33]. In the present study, AKP activity associated with intestinal mucosal barrier function was significantly increased in MR and FR.

The morphological observation of the intestinal tract showed that the intestinal villi of the control group were severely damaged, accompanied by obvious shedding of villus epithelial cells. The KRS diet improved villus damage in the MR group, while the FR group had intact villi. Increased epithelial cell proliferation is one of effects underpinning the beneficial effects of RS on intestinal morphology [34]. Villus length aids the host in breaking down more indigestible carbohydrates [24], which is consistent with our findings.

In the present study, KRS showed benefits in decreasing blood glucose and lipids, indicating its potential to prevent obesity. The ratio of Firmicutes to Bacteroidetes (F/B) corresponds with host obesity, and a high-fat diet reduces the abundance of Bacteroidetes and Actinobacteria [35]. This is in accordance with our findings that KRS reduces the number of Firmicutes while increasing the abundance of Bacteroidetes and Actinobacteria. The species difference test at the genus level revealed that the decline in *norank_f__Mycoplasmataceae* was primarily responsible for the decrease in Firmicute abundance induced by KRS. The *norank_f__Mycoplasmataceae* has positive effects on intestinal tumorigenesis [36,37]. However, the number of Firmicute species such as *Streptococcus*, *Blautia*, *Lactobacillus*, *Eubacterium_hallii_group*, *Clostridium_sensu_stricto_1*, and *Ruminococcus_torques_group* increased in MR and FR. It has been proven that *Streptococcus* and *Eubacterium_hallii_group* can aid with treatment and enhance insulin sensitivity [38,39]. The main species for enteric fermentation and the generation of short-chain fatty acids (SCFAs) are *Blautia*, *Lactobacillus*, *Clostridium_sensu_stricto_1,* and *Ruminococcus_torques_group* [25,40]. Additionally, Actinobacteria and *Bifidobacterium* aid in reducing the prevalence of obesity [41]. Therefore, KRS increased the number of SCFA-producing bacteria in the intestine. Specifically, intestinal SCFAs are mostly composed of acetate, propionate, and butyrate, and activating intestinal SCFA receptors helps prevent fat accumulation [42]. The *Blautia* can produce acetic acid [43], and butyrate synthesis was boosted by bacteria from the Firmicute phyla *Eubacterium_hallii_group*, *Clostridium_sensu_stricto_1,* and *Ruminococcus_torques_group* [44]. Butyrate, a primary source of energy for colonocytes, has several positive effects, including stimulating the de novo formation and expansion of peripheral T-regs, which is required for intestinal homeostasis and barrier function [45].

The function of intestinal bacteria is directly tied to their metabolites, and metabolite profiling can help us to further understand the mechanism of action of KRS. After analyzing the functions of the differential metabolites, we found that they are mainly enriched in metabolic pathways such as amino acid metabolism (particularly arginine production), the metabolism of cofactors and vitamins, carbon metabolism, glutathione metabolism, and the biosynthesis of other secondary metabolites. After resistant starch substitution, the differential metabolites significantly changed in the FR group and were mainly concentrated in amino acid metabolism [24,46,47]. Notably, among the altered metabolites in this study, ten differential metabolites, including 5-hydroxyindoleacetic acid, beta-alanyl-L-arginine, L-glutamine, betaine aldehyde, ornithine, 4-acetamido-2-aminobutanoic acid, L-tyrosine, N-acetylaspartate, nepsilon-acetyl-L-lysine, and N2-acetyl-L-ornithine, were identified as candidate biomarkers for the metabolism of tryptophan, arginine, serine, threonine, tyrosine, lysine, and glycine. The AMPK pathway may be activated by the anabolic process of arginine, which aids in inhibiting the de novo creation of fat [48]. Ornithine, as an arginine metabolite, is positively associated with arginine production and plays a significant metabolic role [49]. The increase in 5-hydroxyindoleacetic acid promotes tryptophan metabolism, which is also beneficial to maintain intestinal homeostasis [50]. Similar to our finding, KRS was shown to have a beneficial effect on enhancing intestinal metabolism by increasing the metabolism of glycine, serine, and threonine in the intestine [51]. In addition, KRS promoted glutathione metabolism and lipid metabolism in the intestine, which may explain the enhanced activity of intestine antioxidant enzymes and decreased serum lipids. In this study, the key metabolites L-glutamine, L-tyrosine, and ornithine are the core metabolites linked to the regulation of different amino acid metabolisms, glutathione metabolism, and lipid metabolism. They are also the key metabolites responsible for the altered intestinal metabolism of KRS. As precursors for arginine synthesis, the decreased intestinal concentrations of L-glutamine and L-tyrosine may be attributed to arginine biosynthesis and metabolism. This also explains the increased concentration of beta-alanyl-L-arginine [52,53].

Based on the results of Pearson’s analysis of intestinal metabolites and microbial composition, the key metabolite was beta-alanyl-L-arginine. The beta-alanyl-L-arginine concentration was positively correlated with *Blautia, Lactobacillus*, *Eubacterium_hallii_group*, *Clostridium_sensu_stricto_1*, and *Ruminococcus_torques_group*. Additionally, *norank_f__Mycoplasmataceae* was proportional to L-glutamine and L-tyrosine concentrations, and spirochaetota was positively associated with fatty acid derivatives including 17-hydroxylinolenic acid, 5,10-pentadecadien-1-ol, and cis-9,10-epoxystearic acid. P-acetaminobenzoic acid and P-acetamidophenol are nitrogen metabolism products in excreta, which explains why proteobacteria improve protein metabolism in the intestine.

Collectively, alterations in intestinal microbiota and metabolite profiles caused by the replacement of common starch with dietary KRS suggest an important role for KRS in the development of intestinal metabolism followed by improved intestinal function and homeostasis.

## 5. Conclusions

In comparison with common starch, KRS effectively improved the intestinal health of hybrid snakeheads. Improved intestinal histomorphogenesis and enhanced intestinal mucosal barriers were observed in the dietary RS group when compared to those in the common starch group. The activities of digestive and antioxidant enzymes in the intestines of KRS group were elevated. Dietary KRS was also found to cause significant changes in the intestinal microbiota and metabolic profile, the majority of which were connected to lipid metabolism and amino acid metabolism. Specifically, dietary KRS raised the abundance of bacteria that can produce SCFAs (especially butyrate) and lowered the abundance of *norank_f__Mycoplasmataceae*. The primary metabolite differentially altered by KRS is beta-alanyl-L-arginine, revealing the potential mechanism of KRS’s roles in preventing obesity, alleviating metabolic syndrome, and improving intestinal barrier integrity. Thus, the findings of this study emphasize the positive influences of KRS on the intestine and provide new insights into the mechanism by which RS improves the intestinal system.

## Figures and Tables

**Table 1:** Experimental diet composition and baseline nutritional content.

Ingredients (%)	C	MR	FR
Fish meal (67%)	35.0	35.0	35.0
Casein	10.0	10.0	10.0
Soybean meal	10.0	10.0	10.0
Lecithin High Potency	1.0	1.0	1.0
Calcium dihydrogen phosphate	1.0	1.0	1.0
Premix *	2.2	2.2	2.2
Choline chloride (50%)	0.2	0.2	0.2
Vitamin C	0.1	0.1	0.1
Fish oil	4.0	4.0	4.0
Microcrystalline cellulose	16.5	16.5	16.5
High-gluten flour	20.0	10.0	
Resistant starch (kelp meal)		10.0	15.0
Binder (KGM, Glucomannan)			5.0
Total	100.0	100.0	100.0
Basic ingredients			
Moisture	7.53	7.43	7.78
Crude protein	39.95	40.41	40.65
Crude fat	6.67	6.75	6.43
Ash	11.01	10.89	11.05

* Premix supplied by Guangdong Bide Biotechnology Co., Ltd., Guangzhou, China. The amounts of following content in per kg of premix are FeSO_4_·H_2_O 10 g, ZnSO_4_·H_2_O 7 g, MgSO_4_·7H_2_O 25 g, CuSO_4_·5H_2_O 0.3 g, MnSO_4_·H_2_O 2.4 g, CoSO_4_·H_2_O 0.02 g, Ca (IO_3_)_2_ 0.4 g, Na_2_SeO_3_, 0.2 g, vitamin A 2,200,000 IU, vitamin B1 4 g, vitamin B2 8 g, vitamin B6 4.8 g, vitamin B12 0.01 g, inositol 40 g, folic acid 1.28 g, biotin 0.064 g, nicotinic acid 28 g, D-calcium pantothenate 16 g, Vitamin C phosphating fat 100 g, vitamin D3 500,000 IU, vitamin E 16 g, vitamin K3 4 g.

## Data Availability

The authors declare that the data supporting the findings of this study are available within the article and its Supplementary Information files.

## Supplementary Materials

The following supporting information can be downloaded at: https://www.mdpi.com/article/10.3390/antiox12081631/s1. Figure S1. Dietary resistant starch modifies the composition of the intestinal microbial population. (A) Chao1 index of OTU levels. (B) Simpson index of OTU levels. (C) Principal Component Analysis (PCoA). (D) Species composition at the phylum level in different groups. (E) Species composition at the genus level in different groups. Values are expressed as means ± SD. * Indicates significant difference (* *p* < 0.05, ** *p* < 0.01, *** *p* < 0.001); Figure S2. Significant variability and functional prediction of intestinal microbes. (A) Significant differences in species at the phylum level. (B) COG functional classification statistical histogram. (C) Significant differences in species at the genus level. (D) COG functional classification statistics box plot. * Indicates significant difference (* *p* < 0.05); Figure S3. KEGG-based pathway prediction. (A) KEGG pathway enrichment of differential metabolites. (A) KEGG network diagram of differential metabolites; Table S1 Information on the origin of feed ingredients; Table S2. Preparation and staining of paraffin sections.
