# Supplementary material for: Microbiome–Metabolomic Analysis Reveals Beneficial Effects of Dietary Kelp Resistant Starch on Intestinal Functions of Hybrid Snakeheads (Channa maculata ♀ × Channa argus ♂)
Source: Antioxidants (Basel). 2023 Aug 18;12(8):1631. doi: 10.3390/antiox12081631 (PMC10451247; doi:10.3390/antiox12081631)
Supplement: Supplementary file 1 [file antioxidants-12-01631-s001.zip › antioxidants-2468834-supplementary.pdf]

Microbiome-metabolomic analysis reveals beneficial effects of dietary kelp resistant starch on intestinal functions of hybrid snakeheads (*Channa maculata* ♀ × *Channa argus* ♂).

Shaodan Wang[1,2], Zhiheng Zuo[2], Bin Ye[2], Li Zhang[1], Yanbo Cheng[1], Shaolin Xie[2], Jixing Zou[2**], Guohuan Xu [1*]

[1]State Key Laboratory of Applied Microbiology Southern China, Guangdong Provincial Key Laboratory of Microbial Culture Collection and Application, Institute of Microbiology, Guangdong Academy of Sciences, Guangzhou 510070, China.

[2]Joint Laboratory of Guangdong province and Hong Kong region on Marine Bioresource Conservation and Exploitation, College of Marine Sciences, South China Agricultural University, Guangzhou 510642, China.

[*]Correspondence: [*]Guohuan Xu, State Key Laboratory of Applied Microbiology Southern China, Guangdong Provincial Key Laboratory of Microbial Culture Collection and Application, Institute of Microbiology, Guangdong Academy of Sciences, Guangzhou 510070, China; E-mail: xghfish@163.com. [**]Jixing Zou, College of Marine Sciences, South China Agricultural University, Guangzhou, 510642, China; Tel/fax: +86-20-87571321; E-mail: zoujixing@scau.edu.cn.

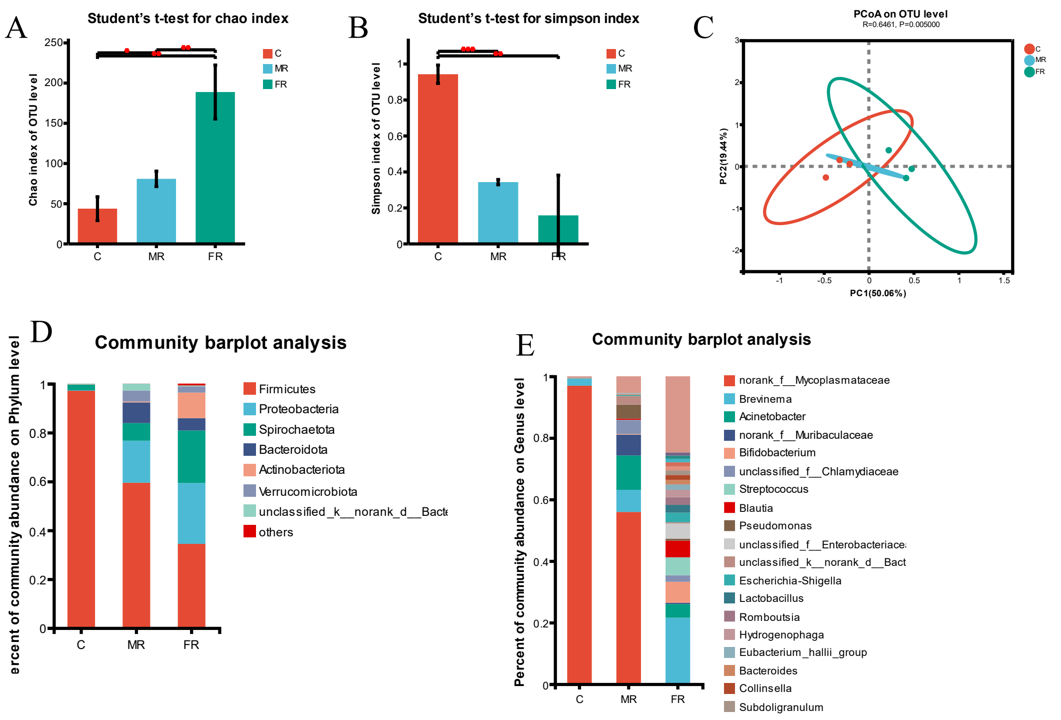

Figure S1 Dietary resistant starch modifies the composition of the intestinal microbial population. (A) Chao1 index of OTU levels. (B) Simpson index of OTU levels. (C) Principal Component Analysis (PCoA). (D) Species composition at the phylum level in different groups. (E) Species composition at the genus level in different groups. Values are expressed as means ± SD. * Indicates significant difference (* $P < 0.05$, ** $P < 0.01$, *** $P < 0.001$).

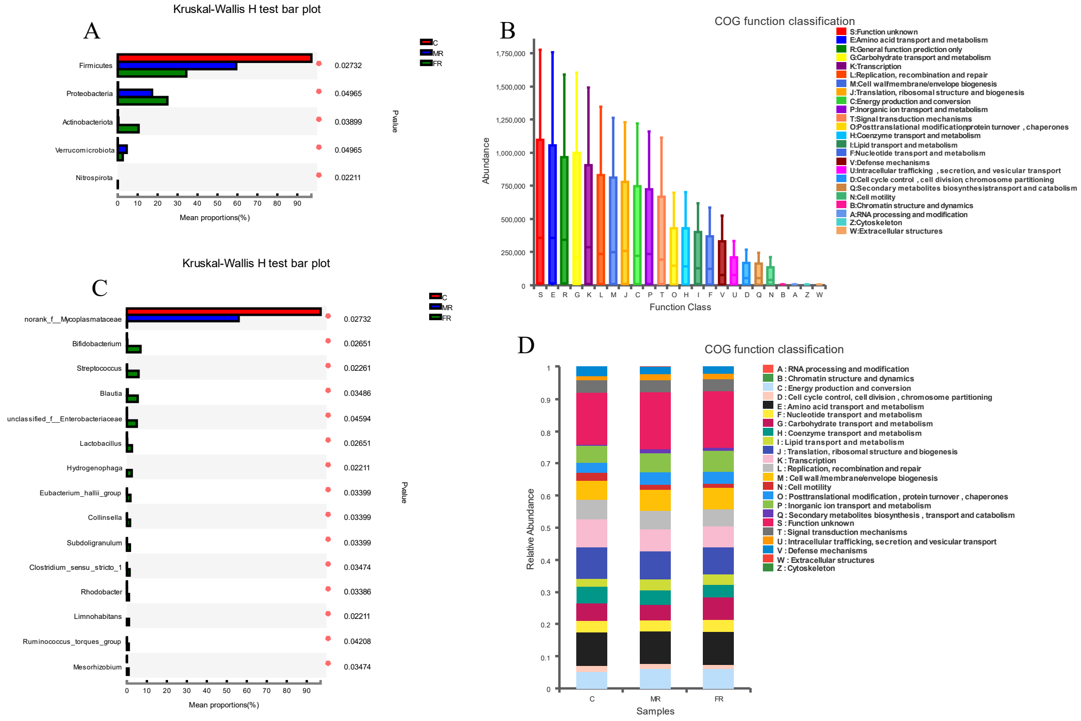

Figure S2 Significant variability and functional prediction of intestinal microbes. (A) Significant differences in species at the phylum level. (B) COG functional classification statistical histogram. (C) Significant differences in species at the genus level. (D) COG functional classification statistics box plot. * Indicates significant difference (* $P < 0.05$).

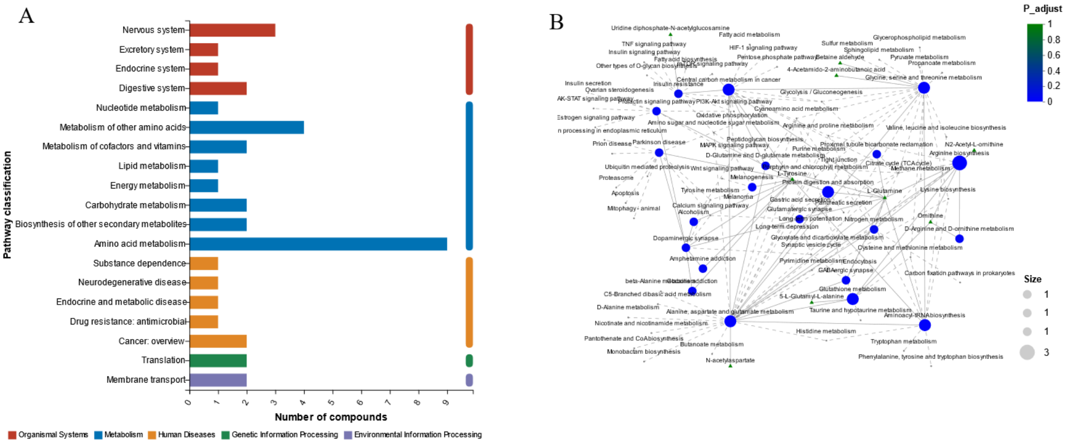

Figure S3 KEGG-based pathway prediction. (A) KEGG pathway enrichment of differential metabolites. (A) KEGG network diagram of differential metabolites.

Table S1 Information on the origin of feed ingredients.

Ingredients	Source
Fish meal (67 %)	New Hope Company (Guangzhou, China)
Casein	Shengruiyuan Biotechnology Co., Ltd (Wuhan, China)
Soybean meal	New Hope Company (Guangzhou, China)
Lecithin High Potency	New Hope Company (Guangzhou, China)
Calcium dihydrogen phosphate	New Hope Company (Guangzhou, China)
Choline chloride (50 %)	New Hope Company (Guangzhou, China)
Vitamin C	New Hope Company (Guangzhou, China)
Fish oil	New Hope Company (Guangzhou, China)
Microcrystalline cellulose	Nuofan Biotechnology Co., Ltd (Wuhan, China)
High-gluten flour	New Hope Company (Guangzhou, China)
resistant starch (kelp meal)	Tiannong Fruit and Vegetable Food Co., Ltd (Xinghua, China)
Binder (KGM, Glucomannan)	Fengbai Biotechnology Co., Ltd (Shandong, China)

Table S2 Preparation and staining of paraffin sections.

Program	Step	Condition
Fixed	Samples were collected and fixed with paraformaldehyde	24 h
Tissue dehydration transparent	75 % Alcohol	4 h
	85 % Alcohol	2 h
	90 % Alcohol	2 h
	95 % Alcohol	1 h
	100 % Alcohol 1st time	30 min
	100 % alcohol 2nd time	30 min
	Alcohol benzene	5-10 min
	Xylene 1st time	5-10 min
	Xylene 2nd time	5-10 min
Wax through	65 °Cmelted paraffin 1st time	1 h
	65 °Cmelted paraffin 2nd time	1 h
	65 °CMelted paraffin 3rd time	1 h
Embedding	Embedding	
Slice	Slice	4 µm
Patch	Smear glycerin protein, scoop flakes	
Baked slices	37 °C Oven	12 h
Section dewaxing and rehydration	Xylene 1st time	20 min
	Xylene 2nd time	20 min
	100 % Alcohol 1st time Alcohol	5 min
	100 % alcohol 2nd time	5 min
	75 % Alcohol	5 min
	Distilled water	3 min
Hematoxylin	Hematoxylin	3-5 min

Program	Step	Condition
staining	staining	
	Rinse with tap water	
	Differentiation fluid differentiation	3-5 s
	Rinse with tap water	
	Back to blue liquid back to blue	5-10 s
	Rinse with tap water	
	85 % Alcohol	5 min
Eosin staining	95 % Alcohol	5 min
	Eosin stain	5 min
	100 % Alcohol 1st time Alcohol	5 min
Dehydrated and transparent slices	100 % alcohol 2nd time	5 min
	100 % Alcohol 3rd time	5 min
	Xylene 1st time	5 min
	Xylene 2nd time	5 min
Mount	Gum	
Baked slices	37 °C Oven	24 h
